# Pharmacogenomics of Lithium Response in Bipolar Disorder

**DOI:** 10.3390/ph14040287

**Published:** 2021-03-24

**Authors:** Courtney M. Vecera, Gabriel R. Fries, Lokesh R. Shahani, Jair C. Soares, Rodrigo Machado-Vieira

**Affiliations:** 1 Experimental Therapeutics and Molecular Pathophysiology Program, Louis A. Faillace, MD, Department of Psychiatry and Behavioral Sciences, McGovern Medical School, The University of Texas Health Science Center at Houston, Houston, TX 77054, USA; Courtney.Vecera@uth.tmc.edu (C.M.V.); Jair.C.Soares@uth.tmc.edu (J.C.S.); 2 Center of Excellence on Mood Disorders, Louis A. Faillace, MD, Department of Psychiatry and Behavioral Sciences, McGovern Medical School, The University of Texas Health Science Center at Houston, Houston, TX 77054, USA; Gabriel.R.Fries@uth.tmc.edu (G.R.F.); Lokesh.R.Shahani@uth.tmc.edu (L.R.S.); 3 Center for Precision Health, School of Biomedical Informatics, University of Texas Health Science Center at Houston, Houston, TX 77054, USA; 4 Neuroscience Graduate Program, The University of Texas MD Anderson Cancer Center UTHealth Graduate School of Biomedical Sciences, Houston, TX 77054, USA

**Keywords:** pharmacogenomics, bipolar disorder, lithium, personalized medicine, predictive models, Genome-Wide Association Study (GWAS)

## Abstract

Despite being the most widely studied mood stabilizer, researchers have not confirmed a mechanism for lithium’s therapeutic efficacy in Bipolar Disorder (BD). Pharmacogenomic applications may be clinically useful in the future for identifying lithium-responsive patients and facilitating personalized treatment. Six genome-wide association studies (GWAS) reviewed here present evidence of genetic variations related to lithium responsivity and side effect expression. Variants were found on genes regulating the glutamate system, including GAD-like gene 1 (*GADL1*) and *GRIA2* gene, a mutually-regulated target of lithium. In addition, single nucleotide polymorphisms (SNPs) discovered on *SESTD1* may account for lithium’s exceptional ability to permeate cell membranes and mediate autoimmune and renal effects. Studies also corroborated the importance of epigenetics and stress regulation on lithium response, finding variants on long, non-coding RNA genes and associations between response and genetic loading for psychiatric comorbidities. Overall, the precision medicine model of stratifying patients based on phenotype seems to derive genotypic support of a separate clinical subtype of lithium-responsive BD. Results have yet to be expounded upon and should therefore be interpreted with caution.

## 1. Background

Despite being the most widely studied mood stabilizer, lithium’s immediate therapeutic mechanism and prophylactic efficacy remain unconfirmed. Lithium, the lightest metal, is a highly reactive monovalent cation with a variety of molecular and neurochemical targets that promote neuroprotection and anti-inflammatory effects in various neurological diseases [1]. It is the first line treatment for Bipolar Disorder (BD) and the most economical mood stabilizer by 10-fold [2], although its efficacy is not the same among all patients. Specifically, estimates generally indicate that 30% of patients are excellent responders while up to 40% fail to respond or experience intolerable side effects [3], including weight gain, acne, renal dysfunction, and thyroid suppression. Over time, lithium use is decreasing despite the drug’s superior anti-manic efficacy and anti-suicidal properties [4,5], perhaps due to the narrow therapeutic window requiring frequent monitoring [6,7].

The ability to predict lithium responsiveness without the arduous process of dose-titration or potential for adverse reactions would be revolutionary for BD treatment. Pharmacogenetic studies are attempting to identify response or tolerability biomarkers, but the process of cherry-picking candidate genes has been proven not to be ideal considering the size of the human genome [2]. Favorable response is more likely for patients with a BD-I diagnosis, few comorbidities, manic/hypomanic-depression-euthymic interval cycle pattern, early age of symptom onset and treatment onset, family history of BD, and adequate drug adherence [8,9,10]. Familial clusters may be a prognosticator for recurrent mood episodes due to patients with good lithium response tending to cluster in families, but alone this datum holds little weight [11]. Another path to genetic predictors is through Genome-wide Association Studies (GWAS), which compares large numbers of patients and controls to identify group differences in common genetic variants. Due to the statistical importance of sample size in GWAS, this article only reviews studies with at least 250 subjects. In contrast to candidate gene studies, GWAS analyses are unbiased in the sense that no a priori hypotheses are defined, which is particularly relevant for multifactorial, polygenic conditions, such as BD. This paper reviews six published GWAS on lithium response in BD, including their main findings, limitations, and perspectives of the field.

### 1.1. Literature Search Criteria

**Figure 1:**
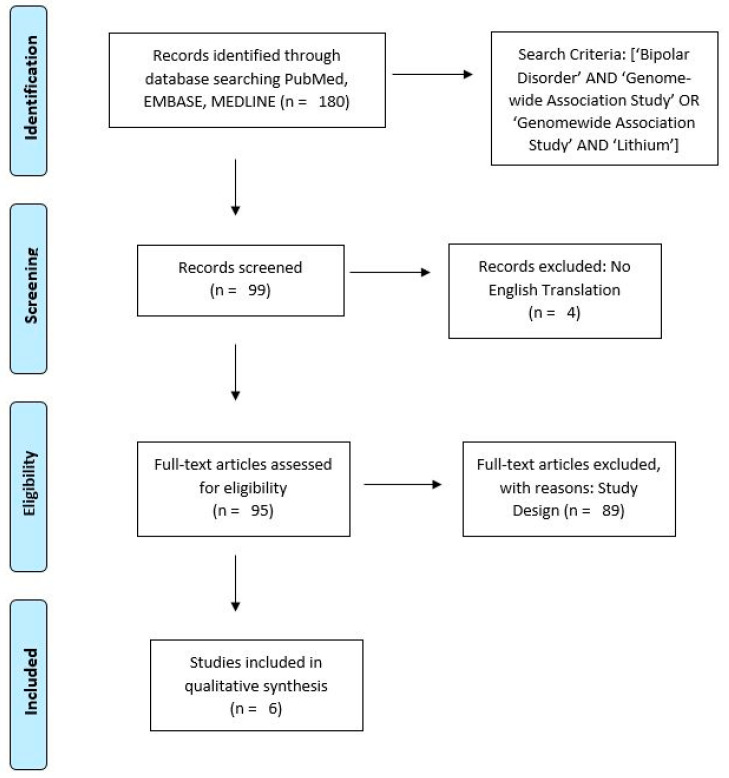
Preferred Reporting Items for Systematic Reviews and Meta-Analyses (PRISMA) Flow Diagram [12].

To identify relevant articles, author CMV searched PubMed, EMBASE, and MEDLINE for articles published between 2000 and 2021 using search terms, “Bipolar Disorder AND (Genome-wide Association Study OR Genomewide Association Study) AND Lithium”. Inclusion criteria were, (1) full text articles in English; (2) sample size of at least 250 subjects; (3) article assessed lithium response in patients with BD (with BD diagnosis based on contemporary Diagnostic and Statistical Manual of Mental Disorders (DSM) criteria), regardless of subtype diagnosis. Review articles were excluded. Re-analyses of GWAS datasets using complementary approaches (such as cross-trait analysis) were eligible for inclusion. 180 articles were initially identified; after duplicates and articles without English translations were removed, 95 full-text articles were screened. Six articles were considered eligible and were included in the review. Figure 1 presents the results of the literature search identifying the six GWAS articles used in this review.

### 1.2. Definitions of Response

For approximately one-third of BD patients, lithium pharmacotherapy prevents mood episodes and reduces BD-related functional impairment and symptoms, including suicidality [5,13,14]. The GWAS articles reviewed here have been inconsistent regarding phenotypic definitions of lithium response. Perlis and collaborators and Song and collaborators used pre- and post-treatment psychometric measures, while the other four studies used the Alda scale to evaluate long-term lithium response. The Alda scale (Retrospective Assessment of the Lithium Response Phenotype Scale) is a validated index that uses two criteria to first define an association between the treatment and clinical improvement on a continuous scale (0–10), then to establish causation on an ordinal/dichotomous scale [15]. Together, these criteria measure the change in illness episodes (frequency, duration, severity) over the course of lithium treatment and quantify symptom improvement [11,15]. See Table 1 for an overview of the six selected GWAS reviewed here.

**Table 1:** Overview of Selected Genome-wide Association Studies (GWAS).

Reference	Sample Size	Definition of Response	Key Findings	Significance of Findings
Perlis et al. [16]	458 discovery, 359 replication	Pre- & post-treatment interviews/measures	SNPs on Ch 4, *GRIA2* gene region	Significant in case-control analysis
Chen et al. [17]	294 discovery, 100 replication, 24 follow up	Retrospective Alda Scale	SNPs on Ch 3, *GADL1* gene	Genome-wide significance
Song et al. [18]	3874	Pre- & post-treatment interviews/measures	SNP on Ch 2, *SESTD1* gene	Significant in case-control analysis
Hou et al. [19]	2563 discovery, 73 replication	Retrospective Alda Scale	4 SNPs on Ch 21, lncRNA *AL157359.3* & *AL157359.4* genes	Genome-wide significance
ConLiGen [20]	2586	Retrospective Alda Scale	Inverse association of SZ PGS and lithium response; Variants in HLA Antigen genes	Direction was not statistically significant; Genome-wide significance
Amare et al. [21]	2586	Retrospective Alda Scale	Inverse association of MD PGS and lithium response	Significant only in European cohort

## 2. GWAS of Lithium Response in BD

In 2009, Perlis and colleagues conducted the first GWAS of lithium response in patients with BD. The five-year study examined associations for risk of recurrent mood episodes in 1177 BD patients, 458 of whom received lithium carbonate or citrate. They did not differentiate between monotherapy and adjunctive drugs, nor between BD subtype diagnoses, a translational approach that sacrificed precision. All participants had Western European ancestry and lived in the United Kingdom (UK) or Ireland. The study used naturalistic conditions without masking to compare lithium’s prophylactic efficacy with that of other mood stabilizers. GWAS and a replication analysis in a second cohort of 359 BD patients on lithium tested the single nucleotide polymorphisms (SNPs) with the greatest significance. Based on Grof and collaborators [11], Perlis and collaborators defined lithium response as two or fewer mood symptoms over eight weeks. Relapse was defined based on the DSM-IV nosography [16].

When comparing responders and non-responders, the authors found no significant genomic associations with lithium response. A subsequent analysis comparing responders with healthy controls found several SNPs in the glutamate ionotropic receptor AMPA type subunit 2 (*GRIA2*) gene, which codes for ionotropic AMPA (α-amino-3-hydroxy-5-methyl-4-isoxazolepropionic acid) receptor’s GluR2 subunit. According to Seelan and collaborators, 2008, *GRIA2* expression is regulated by acute lithium treatment. Specifically, chronic lithium use has been shown to downregulate this gene in vitro and reduce hippocampal GluR2 synaptic expression [22,23]. Glutamate has been implicated extensively in mood disorder pathophysiology, and magnetic resonance spectroscopy (MRS) imaging has shown increased hippocampal glutamate concentrations in euthymic patients on long-term lithium treatment in relation to healthy controls [24]. Chronic lithium use at therapeutic doses has also shown neuroprotective properties against glutamatergic excitotoxicity via NMDA (*N*-methyl-d-aspartic acid) receptor mediated calcium inflow [25].

Song and colleagues (2016) performed a GWAS on a sample of 3874 BD lithium-users from Sweden and the UK, 323 of whom were objectively good responders based on clinical documents, and 1639 who self-reported good lithium response. Lithium response was treated as a dichotomous phenotype (i.e., responder or non-responder) determined through subjective and objective psychometric assessments, patient interviews, and chart reviews. The heterogeneous sample was stratified based on nationality and phenotype. Following association tests, cases were pooled and re-stratified for a case-control meta-analysis. The first analysis, responders versus non-responders, yielded no SNP of genome-wide significance in either population. The case-control analysis saw two SNPs of significance emerge from objective measures, rs146727601 and rs116323614, but only the latter reached genome-wide significance. Located on 2q31.2 in an intron of SEC14 and spectrin domains 1 (*SESTD1*), rs116323614 was successfully validated as a correlate of lithium response with an estimated SNP heritability rate comparable to the heritability estimates of BD (h^2^ = 0.25–0.29) [18,26].

*SESTD1* encodes a synapse protein that directly binds to phospholipids. It facilitates cell membrane turnover, intracellular protein transport, and phospholipid signaling protein assembly [27]. Interactions with *SESTD1* may contextualize lithium’s exceptional ability to cross cell membranes and penetrate the blood–brain barrier (BBB). Of note, *SESTD1* may mediate lithium’s effects on inositol phospholipids, as several lines of evidence implicate inositol phospholipids in BD pathophysiology and as a target of lithium [28,29]. Although its specific function in humans is unclear, a preclinical study has determined it an essential gene, as *SESTD1* knockout (KO) mice die within a day of birth [30]. Past GWAS have implicated *SESTD1* variants in a variety of abnormal phenotypes affecting uric acid levels, renal disease [31], and antiphospholipid antibodies [32,33]. This suggests that this variant is expressed in multiple simultaneous phenotypes, likely with multiple alleles each [18].

Chen and colleagues studied a homogeneous BD sample with Han Chinese ancestry. Researchers selected cohorts from a sample of 1761 BD-I patients receiving long-term lithium monotherapy, excluding those with affective or psychotic comorbidities [17]. The first cohort included 294 medication-adherent patients as part of the discovery stage, during which researchers identified regions of the genome associated with lithium response. Response was defined using the Alda scale [11,15,34]. The association analysis and subsequent follow up study identified two SNPs of genome-wide significance, rs17026688 and rs17026651, located in the introns of a gene encoding glutamate decarboxylase-like protein 1 (*GADL1*). Using distinct samples with the same criteria, tests of replication further corroborated the variants and predicted lithium response with 93% sensitivity [17].

The GADL1 protein belongs to the Group II decarboxylase family, expressed broadly in the human brain. The protein’s functional mechanism is unclear, but based on similarities to Glutamate Decarboxylase (GAD), may involve GABA (γ-aminobutyric acid) synthesis and increasing neural inhibition [35]. These findings underscore the importance of the glutamatergic system in lithium response [17], similar to those reported by Perlis and collaborators [16]. Accordingly, chronic lithium use has been shown to alter glutamate receptor distribution and synaptic morphology [36].

In this same study [17], researchers concluded that nearly half of their subjects carried the rs17026688 response allele predicting favorable lithium response, a considerably higher frequency than is seen in the general population [37]. This may suggest that the allele is more generally linked to BD risk than lithium response, but further examination is merited. These hint to lithium response biomarkers in BD-I patients of Han Chinese-descent. The associated alleles are uncommon in people with African or European descent, so their use may be limited by population stratification and does not explain the full genetic influence on lithium response. Other *GADL1* variants in complete linkage disequilibrium with rs17026688 have also emerged as potential influences on mRNA splicing, further supporting the unique lithium responder phenotype [17]. However, three subsequent studies failed to replicate Chen and colleagues’ findings in a Caucasian sample [38], Indian sample [39], and in vitro with tissue from Caucasian patients [40].

In 2016, Hou et al. set out to identify genetic variants associated with lithium response among BD-I and II patients, analyzed in separate cohorts based on ancestry. Data was collected on 2563 patients from 22 sites as part of the International Consortium on Lithium Genetics (ConLiGen) database and rated on the Alda scale. Genotyping revealed a significant genome-wide association between lithium response and four SNPs on chromosome 21 (rs79663003, rs78015114, rs74795342, and rs75222709). The lithium response-associated chromosomal locus of the four SNPs contains genes for two long, non-coding RNAs (lncRNA), *AL157359.3* and *AL157359.4*. In the central nervous system (CNS), lncRNA play an important role in gene regulation, among other functions that could help explain lithium’s genetic mechanisms [19].

This SNP cluster showed strong linkage disequilibrium, one indication of causation for at least one identified variant, though further analysis is warranted. Variants had similar minor allele frequencies, supporting a hereditary model of lithium-responsiveness. Importantly, prior GWAS show that common alleles generally produce more substantial effects on pharmacogenomics traits than individual variations [41,42]. To test whether the relationship was confounded by overall genetic risk of BD, researchers tested for correlations between risk profiles and Alda scores, detecting none. A subsequent prospective study with an independent sample of 73 BD patients on lithium monotherapy corroborated these results. Specifically, carriers of the alleles associated with poor response to lithium indeed showed higher rates of relapse than non-carriers. Hou and colleagues’ results support a polygenic phenotype of lithium responsivity in BD. If confirmed by larger prospective studies, these data could have direct clinical applicability as one part of a lithium response PGS screening tool [19].

In a separate, more recent study, ConLiGen researchers investigated the relationship between lithium responsiveness and genetic susceptibility for schizophrenia among 2586 BD patients undergoing long-term lithium pharmacotherapy. Sampled were 2366 patients with European ancestry and 220 patients with Asian ancestry. The multi-stage study utilized cross-trait meta-analysis and pathway analysis to identify and characterize individual genetic variants and their associated molecular origins. This study presented two main findings. First, an inverse association between genetic loading for schizophrenia risk variants and long-term lithium response. Second, a potential role for the HLA complex and inflammatory cytokines in BD lithium treatment response [20].

Using PGS, the same study revealed a strong correlation between lower polygenic load for schizophrenia and lithium responsiveness across Alda scale phenotypes [20]. This is consistent with past findings implicating an inverse relationship between lithium response and severity of psychotic symptoms in BD [43]. Even BD patients with family history of schizophrenia show markedly poorer response to lithium [34]. Despite or perhaps because of this relationship, lithium is not an efficacious treatment for schizophrenia. In fact, in these populations, lithium can show neurotoxic effects even at subclinical doses [44].

Network analyses indicate tumor necrosis factor (TNF), interleukin-4 (IL-4), and interferon-γ (IFNγ) as predominant functional nodes in the relationship between lithium response and schizophrenia genetic predisposition [20]. Follow up functional analyses of these loci revealed highly represented canonical pathways, including the antigen presentation pathway, OX40 signaling pathway, B-cell development, Cdc42 signaling pathway, and autoimmune thyroid disease signaling. Together these pathways play an important role in immune function, modulation of inflammatory cytokines, and cellular/intracellular communication. Future studies should target these paths as potential predictors of lithium tolerability or side effect severity. For clinical translation, these findings may be combined with other genetic, biological, and clinical markers to predict BD lithium treatment response since the polygenic load for schizophrenia accounted for just 1% of actual variation in response and has yet to be replicated [20].

Building on findings from the previous study, Amare and colleagues (2020) assessed a connection between depression and lithium response [21]. Comparing summary statistics from a GWAS in Major Depression (MD) (135,458 cases and 344,901 controls) to the 2586 BD patient ConLiGen sample, they determined response using the Alda scale. Using a parallel study design with the same BD sample, a later GWAS successfully replicated the association [21].

Some clinicians find lithium more therapeutically effective against manic episodes than depressive ones [2]. In parallel, Amare and collaborators found that BD patients with higher genetic loading for MD tended to be less responsive to lithium therapy than those with lower MD-PGS. This finding was statistically significant in the combined sample, but when cases were stratified by ethnicity, the correlation was robust in the European cohort, but only marginal in the smaller Asian-ancestry cohort. PGS analyses of MD-related genetic variants indicated that BD patients with low genetic predisposition to MD were 150% more likely to be good lithium responders. Based on the self-reported number of depressive episodes, a significant positive correlation emerged suggesting that higher genetic loading for MD may exemplify a distinct BD phenotype characterized by frequent, severe depressive episodes [21].

In contrast, family studies have failed to link lithium response in BD with MD genotypes or family history of MD [34]. Lithium’s well-documented efficacy as an adjunctive therapy for treatment-resistant depression also appears contradictory [45]. The authors posit that lithium’s antidepressant mechanism is disparate from its actions in long-term BD treatment. The genotypic and phenotypic overlap between BD and MD may provide a mechanistic explanation for the group’s findings [46], but further study is necessary. The inverse relationship between lithium response and genetic loading in MD and BD (as shown by the group’s previous study) not only supports a polygenic determination of response, but may also illustrate a larger trend between comorbidity and treatment response in BD [20,21]. Specifically, poor BD treatment outcomes are associated with comorbid anxieties [47], substance use/addiction [48], personality disorders, and autoimmune, metabolic, allergic, and thyroid disease [49]. However, these diseases are also extremely prevalent in BD populations, complicating treatment further [10].

**Figure 2:**
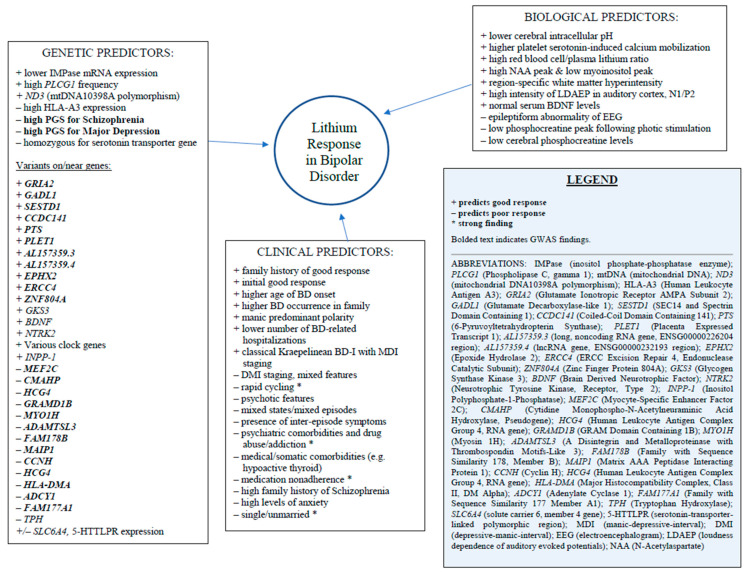
Genetic, Biological, and Clinical Predictors of Lithium Response in Bipolar Disorder [5,8,9,10,11,15,16,17,18,19,20,21,40,44,49,50,51,52,53,54,55,56,57,58,59,60,61,62].

The stratification of lithium-responsive BD as a separate phenotype characterized by more manic features and less psychiatric comorbidity may be useful for future research, but should be interpreted with caution since only approximately 1% of genetic variations in lithium response can be traced to MD-PGS. Therefore, these markers alone have little clinical value, but combined with other biomarkers, may be more translational [21]. See Figure 2 for an overview of known genetic, biological, and clinical predictors of lithium response in BD.

## 3. Limitations

As GWAS of complex and heterogeneous phenotypes like BD rely on sample size for statistical power and accuracy, all studies reviewed here were limited by small sample sizes. Although most included subsequent replication analyses, more data derived from rigorous, controlled clinical studies will be essential to confirm results and achieve clinical utility. A vital aspect of pharmacogenomic research involves implementing universal guidelines for diagnosis and drug response. While all groups took efforts to ensure accuracy of diagnostic procedures, methods and definitions of response varied widely. Three studies used the Alda scale, which only provides retrospective data susceptible to recall bias and missing or inaccurate data. Additionally, the scale does not evaluate phase changes, illness course, or neurobiological/ pathophysiological changes. This is also the case for the three studies that recruited from the same diverse ConLiGen sample, trading precision for real-world applicability. Homogeneity in population stratification was another limitation. Chen et al. in particular used an extremely uniform sample which resulted in startling findings that have never been replicated. Until the findings from these GWAS are adequately replicated in prospective studies, the direction of the associations between SNPs and lithium response cannot be determined nor assigned causality.

Another major limitation in the GWAS and BD research more broadly is underrepresentation of minority populations. The lack of GWAS in diverse and minority populations likely reflects missed opportunities in understanding racial and ethnic differences in Bipolar Disorder and BD treatment response [63]. In fact, a randomized controlled trial found that compared to Caucasians, African Americans with BD showed significantly better antidepressant response and greater improvements in quality of life on low-dose lithium, but no pharmacogenomics studies have attempted to examine what should be a remarkable finding [64]. A comprehensive review of racial disparities in BD diagnosis, treatment, and research participation found consistent evidence of systemic racial biases that predominantly affected patients of African ancestry in all three areas [63].

## 4. Clinical Applications for Precision Medicine

Considering that BD is a multifactorial disease, possible environmental interactions with genomic predictors should not be overlooked. Recently, Marie-Claire and colleagues found potential epigenetic signatures of lithium response by examining whether distinct blood DNA methylation profiles correlated with prophylactic lithium response in a small sample of BD-I patients [65]. A genome-wide analysis comparing excellent lithium responders with non-responders revealed 111 differentially methylated regions (DMR) associated with significant neuronal cell enrichment that support lithium’s role in neuroprotection and promoting neurogenesis [1,50]. The group also identified seven DMRs able to differentiate between responders and non-responders with excellent discriminative power (area under the curve/AUC above 0.8). Results suggest that biomarkers of lithium response may be detected via peripheral epigenetic markers, such as methylation profiles. The study had several significant limitations, particularly related to an inability to control for various influences on methylation status, from BD disease progression to pre-existing genetic and environmental differences, but presents promising preliminary findings that merit replication with a larger sample and prospective study design [65].

BD treatment involves stabilizing mood, preventing future mood episodes, and curbing dysfunction. However, medical and psychiatric comorbidities can significantly disrupt treatment, necessitating more expensive and time-consuming options that are not feasible for many patients. Screening tools or prognostic algorithms comprised of several different components (neuroimaging and blood biomarkers, clinical features and demographic data, genetic variants, and additional diagnoses) may be able to more accurately predict treatment response and tolerability [10]. This would allow clinicians to eliminate many of the roadblocks to BD remission: inaccurate/untimely diagnosis, experiences with severe side effects, and the arduous process of dose titration.

An important part of a personalized approach to treatment is research. Stratifying patients based on different phenotypes and genotypes during replication studies and future GWAS will allow for more patients to be represented. Stone and colleagues recently tested a unique machine learning approach to genomic classification of lithium response in BD [66]. Researchers used genomic data from 2210 BD patients (from the ConLiGen database) based on 47,465 SNPs to predict lithium response at above-chance levels and with relatively higher specificity than sensitivity. Importantly, findings suggest that prospective approaches yield better predictability than retrospective Alda score data, highlighting the limitations of several GWAS reviewed here [66].

The six studies reviewed here provide varying degrees of evidence in support of patient stratification and the importance of biological differences in treatment. Precision medicine aims to tailor patient care through pharmacogenomic/pharmacogenetic approaches that will likely still be in development for years, and in ways possible now. For example, autoimmune thyroid disease comorbid to BD is generally deemed unfit for lithium therapy, despite the drug’s superior prophylactic efficacy. A personalized approach might be to prescribe lithium with adjunctive T3 hormones to accelerate lithium response and protect against thyroid suppression while maintaining lithium’s neuroprotective effects [67]. Increasing resilience over time while targeting behavioral problems, providing psychoeducation, and utilizing pharmacotherapies together are more effective than any one approach.

## 5. Conclusions

Six GWAS concerning lithium response in BD were reviewed in this article. Variants associated with lithium response were found on genes regulating the glutamate system, including *GADL1* and *GRIA2* genes [16,17]. SNPs discovered on *SESTD1* may account for lithium’s exceptional ability to permeate cell membranes and mediate autoimmune and renal effects [18]. Studies reviewed here also corroborated the importance of epigenetics and stress regulation on lithium response, finding variants on lncRNA genes and in HLA genes associated with response to lithium [19,20]. Additionally, study findings implicate an inverse relationship between lithium response in BD and genetic risk for Schizophrenia and Major Depression [20,21]. Because pharmacogenomics is only in its infancy in psychiatry, individual findings are unlikely to hold translational significance if not replicated by independent cohorts. Together, they begin to paint a picture of heritable genetic influences passed down through ancestry that modulate the heterogeneous lithium response seen in clinical settings. Response and side effect presentation are likely determined by multiple genes, many of which have multiple alleles, as well as epigenetic and environmental influences. GWAS data supports patient stratification in research and diagnosis to aid precision medicine. Lithium response may be seen as a separate phenotype with coinciding genomic variations. A better understanding of lithium and of the human genome would facilitate this approach.
